# A Case Study of a Rare Disease (Fructosemia) Diagnosed in a Patient with Abdominal Pain

**DOI:** 10.3390/jcm13123394

**Published:** 2024-06-10

**Authors:** Leszek Garbowski, Marzena Walasek, Rafał Firszt, Ewelina Chilińska-Kopko, Paulina Błażejewska-Gała, Daniel Popielnicki, Zofia Dzięcioł-Anikiej

**Affiliations:** 1 Public Independent Healthcare Services of the Ministry of Internal Affairs and Administration in Białystok, 15-471 Białystok, Poland; 2 Department of Human Anatomy, Medical University of Białystok, 15-089 Białystok, Polandpaulina.blazejewska@umb.edu.pl (P.B.-G.); danilapopelnicki@gmail.com (D.P.); 3 Department of Ornamental Plants and Garden Art, Faculty of Biotechnology and Horticulture, University of Agriculture in Krakow, 31-425 Kraków, Poland; rafal.fir@gmail.com; 4 Department of Neonatology and Newborn Intensive Care, University Clinical Hospital in Białystok, 15-276 Białystok, Poland; 5 Department of Rehabilitation, University Clinical Hospital in Białystok, 15-276 Białystok, Poland

**Keywords:** hereditary fructose intolerance, fructosemia, hypoglycemia, fatty liver, growth retardation, aldolase B

## Abstract

Hereditary fructose intolerance is a rare genetic disorder that is inherited in an autosomal recessive manner, with mutations sometimes occurring spontaneously. Consuming fructose triggers biochemical abnormalities, disrupting liver processes like glycogenolysis and gluconeogenesis. Recent studies have revealed elevated intrahepatic fat levels in affected individuals. Symptoms include aversion to fructose-containing foods, hypoglycemia, liver and kidney dysfunction, and growth delays, with severe cases leading to liver enlargement, fatty liver disease, kidney failure, and life-threatening hypoglycemia. In this case study, we present a 20-month-old child with symptoms including difficulty passing stool, abdominal rigidity, abdominal pain with bloating and hypoglycemia. Initial clinical findings revealed elevated liver enzymes, a mildly enlarged hyperechoic liver, hypercholesterolemia, and borderline alpha-fetoprotein values. Diagnostic assessments identified hereditary fructose intolerance (HFI) with pathogenic variants in the ALDOB gene, along with a diagnosis of celiac disease. Genetic testing of the parents revealed carrier status for pathological aldolase B genes. This case underscores the importance of comprehensive clinical evaluation and genetic testing in pediatric patients with complex metabolic presentations.

## 1. Introduction

Hereditary fructose intolerance is a rare genetic disorder. It is inherited in an autosomal recessive manner, with mutations occasionally occurring de novo. It is classified as a rare disease, with its prevalence ranging from 1:20,000 to 1:60,000. Hereditary fructose intolerance affects both sexes equally [1].

Fructose is a monosaccharide that is abundant in fruits, honey, many vegetables, and highly processed foods. It is also produced in small amounts in the human brain via the polyol pathway [2]. Upon consumption, fructose is absorbed from the intestine via glucose transporter proteins (GLUT) 5 and 2. Subsequently, metabolism occurs primarily in the liver, kidneys, and small intestine, involving enzymes such as fructokinase, aldolase B, and triokinase [3].

In patients with aldolase B deficiency, consumption of fructose, sucrose, or sorbitol triggers a sequence of biochemical abnormalities, leading to short- and long-term consequences of that disease. Intracellular trapping of phosphates and accumulation of fructose-1-phosphate [Fru 1P] are responsible for disturbances in biochemical processes occurring in the liver: glycogenolysis and gluconeogenesis are inhibited. Hypoglycemia, especially postprandial, arises from those abnormalities after fructose consumption. Hypoglycemia may be severe and life-threatening for a patient [4]. 

The decreased ATP (adenosine triphosphate) levels alter the AMP (adenosine monophosphate): the ATP ratio, leading to an increased AMP breakdown, results in hyperuricemia [5]. Elevated uric acid levels may cause gout symptoms. Hypophosphatemia and accompanying hypermagnesemia are also observed [6]. 

If untreated, the disease leads to the dysfunction of the liver and kidneys. In patients with fructosemia, liver fibrosis is observed. Renal changes include a syndrome similar to Fanconi syndrome and nephrocalcinosis. The mechanism underlying the renal dysfunction is not well defined [7].

One of the most typical symptoms of fructosemia is a natural aversion to products containing fructose—patients have an aversion to the consumption of fruit, juice, fruit and vegetable purees, as well as most sweets. Due to this natural aversion to certain foods, their symptoms of fructose intolerance decrease. The low intensity of fructose intolerance symptoms or their absence may mean that a diagnosis of fructosemia is delayed until adulthood [8].

A newborn does not manifest any symptoms when consuming their mother’s breast milk. Characteristic symptoms such as vomiting, nausea, and excessive sweating, which are associated with hypoglycemia and metabolic acidosis, appear after the child is switched to powdered milk sweetened with sucrose or has their diet extended to include foods containing sugar as well as fruit and vegetables. If a large amount of fructose is consumed, the reaction becomes more intense. The infant becomes apathetic and sleepy. Seizures may occur [9].

In the case of a prolonged consumption of a high-fructose diet, progressive liver damage may develop, leading to its enlargement and steatosis as well as to NAFLD (non-alcoholic fatty liver disease) [10]. 

There is also a noticeable growth delay in patients compared to their peers [11].

## 2. Patient Information

In February 2023, Caucasian parents arrived at the Family Healthcare Clinic with their 20-month-old child. They sought a medical consultation with regard to the child’s abdominal rigidity and difficulty passing stool. A week earlier, the child had had an episode of abdominal pain with bloating. Until the medical consultation, the child had passed stool regularly. The medical treatment included macrogol and lactulose.

The child was born from a second pregnancy complicated by maternal hypothyroidism (the first pregnancy ended in a miscarriage). The delivery took place at 39 weeks’ gestation through a cesarean section due to fetal heart dysfunction. Despite those complications, the child scored 10 points on the Apgar scale at 1 min after birth. During the newborn’s physical examination, excessive hair growth was noted around the auricles and in the lumbosacral region. The child experienced hypoglycemia on their third day of life, which was treated with intravenous infusion of 10% glucose.

During a visit at the GP’s office, a physical examination indicated abdominal distension, with elevation above the level of the chest. The auscultation of the lung fields revealed normal vesicular breath sounds, with a steady heart rate of approximately 84/min. Peripheral lymph nodes were not enlarged.

The laboratory screening tests performed on the day of examination showed elevated alanine aminotransferase (ALT) activity (95 U/L) (refer to Table 1 for comprehensive laboratory test results). Upon receipt of the results, abdominal ultrasound sonography, additional tests (total bilirubin, aspartate aminotransferase (AST), gamma-glutamyl transferase (GGTP), creatinine, stool examination for parasites, creatine kinase), macrogol administration, and increased oral hydration were recommended urgently.

During the follow-up visit (three days later), the ALT was 100, the AST was 99, and the GGTP was 64 IU/L. The other measured parameters were normal. The abdominal ultrasound sonography revealed a hyperechoic, mildly enlarged liver. The child was referred to the Paediatric Department for further diagnostic assessment and treatment.

During a 7-day hospitalization at the Paediatric Gastroenterology Clinic, elevated liver enzyme activity (AST 84 U/L, ALT 78 U/L, GGTP 56 IU/L) and the enlarged hyperechoic liver were observed again. Hypercholesterolemia (total cholesterol 215 mg/dL) and borderline AFP (alpha-fetoprotein) values of 8.38 ng/mL were also noted. The child felt well during the hospitalization, and stool passage normalized. Upon discharge, the child was referred to the Metabolic Diseases Clinic and monitoring of their liver enzymes was advised.

One week after the hospitalization, the elevated liver enzyme activity persisted (ALT 132, AST 100, GGTP 67). A follow-up abdominal ultrasound sonography revealed enlarged hyperechoic liver once again.

A visit to the Metabolic Diseases Clinic took place in April 2023, suggesting the possibility of fructose metabolism disorders. The child was scheduled for admission to the Paediatric Metabolic Diseases Clinic (June 2023). During the hospitalization, blood was drawn for fructosemia testing. Additionally, from separate samples, total IgA, IgA antibodies against tissue transglutaminase (IgA-tTGA), and IgA antibodies against endomysium (EMA-IgA) were measured, yielding a result sufficient for the diagnosis of celiac disease. IgA-tTGA exceeded the upper limit of normal (>10 times) (158.1 U/mL), allowing small intestine biopsy to be avoided.

The molecular testing revealed the presence of a pathogenic variant c.448G > C p.Ala150Pro{A150P} and a pathogenic deletion c.419_422del p.(Lys140Metfs*12), confirming the diagnosis of hereditary fructose intolerance. Those mutations resulted in the residual enzyme activity involved in the fructose metabolism.

In September 2023, genetic testing was also conducted on the child’s parents. During the genetic consultation, it was found that the parents, who are unrelated and without a history of other diseases, are carriers of pathological aldolase B genes. The DNA sequencing analysis using the Sanger method revealed a mutation in ALDOB: c.419_422del/- in the mother and ALDOB: c.448G > C/- in the father.

## 3. Diagnostics and Treatment

The symptoms of hereditary fructose intolerance may vary in terms of severity, depending on the residual activity of aldolase B enzyme. Many of the symptoms are nonspecific and also occur in other health conditions. This may lead to the disease remaining undiagnosed for years [12].

Abnormal fructose metabolism may be indicated by deviations in the urine analysis (fructose detection). Also blood tests may reveal low glucose and phosphate levels as well as elevated magnesium, uric acid, and lactic acid (lactic acidosis). However, those findings are not pathognomonic for that disease [13]. During the hospitalization of the patient under consideration, no elevated fasting glucose levels (83 mg/dL) were observed, and the acid–base balance (ABB) was kept within the reference range, whereas the serum inorganic phosphate was slightly above the reference value (6.01 mg/dL).

The genetic, autosomal recessive nature of the disease should prompt the attending physician to review the patient’s family history. Attention should be paid to cases of diagnosed fructosemia and unexplained liver and kidney failures, which may suggest fructosemia and guide proper diagnostics, although they do not confirm the diagnosis. The absence of the aforementioned health conditions in family members does not exclude the presence of fructosemia [14].

For the purpose of diagnosing fructosemia, a fructose loading test may be used. It is rarely used because fructose loading significantly increases the risk of severe hypoglycemia in patients. Liver biopsy is also possible; however, as it is an invasive procedure, genetic testing is recommended to confirm the diagnosis instead of measuring aldolase B activity in liver biopsy samples. Genetic tests are highly effective. They include single-gene sequencing, multi-gene panels, and genome-wide association studies [15].

The aldolase B enzyme gene (ALDOB) is situated on chromosome 9q22.3. Mutational aberrations encompass simple missense mutations, deletions, frameshift mutations, and splice-site mutations. A systematic review was conducted to evaluate ALDOB gene variants among patients with hereditary fructose intolerance (HFI). The systematic review comprised 1426 alleles implicated in the pathogenesis of HFI, which were distributed across 29 countries on four continents [10]. Among HFI patients from diverse populations, 68 ALDOB variants were identified. These variants were detected in 85 different genotypic combinations. The most widely distributed variants globally, constituting the majority of identified cases, include the following:

(1) NM_000035_3:c.178C > T, NP_000026.2: p.(Arg60Ter).
(2) NM_000035_3:c.360_363del, NP_000026.2: p.(Asn120LysfsTer32).
(3) NM_000035_3:c.448G > C, NP_000026.2: p.(Ala150Pro).
(4) NM_000035_3:c.524C > A, NP_000026.2: p.(Ala175Asp).
(5) NM_000035_3:c.1005C > G, NP_000026.2: p.(Asn335Lys).

Analyses demonstrated that the most prevalent variants among patients are p.(Ala150Pro) and p.(Ala175Asp), accounting for approximately 68% of alleles. The p.(Ala150Pro) variant alone represents 53% of all identified alleles worldwide and exhibits variable frequencies across different geographical regions. The p.(Asn120LysfsTer32) variant is the third most frequent (4.6%) [5,16,17].

In the described case of a 20-month-old child, sequencing analysis of exon 5 and adjacent intronic sequences were conducted to ascertain the presence of common pathogenic variants responsible for fructose intolerance, occurring in the investigated regions of the ALDOB gene. The identified variant c.419_422del p.(Lys140Metfs*12) in the ALDOB gene is a novel variant, involving a deletion of four nucleotides, which induces a frameshift in the protein reading frame and likely results in its truncation, potentially leading to protein or transcript degradation [16].

Recent studies conducted on aldolase B-deficient mice and patients with HFI have shed new light on the pathogenesis of HFI, particularly its impact on the liver. Both groups, when restricting fructose intake, exhibited higher levels of intrahepatic fat compared to control groups. Genetic modifications in aldolase B-deficient mice reduced intrahepatic Fru 1P concentrations, preventing the associated liver phenotype. Magnetic resonance imaging confirmed elevated intrahepatic triglyceride levels in HFI cases, partly due to increased de novo lipogenesis in aldolase-deficient mice. These findings suggest potential treatments for HFI, emphasizing the role of fructose metabolism and its implications for liver health [18,19,20,21].

As hereditary fructose intolerance (HFI) is a multifaceted metabolic disorder, its management necessitates a multidisciplinary approach involving pediatricians; clinical geneticists; dietitians specializing in metabolic disorders; hepatologists; and nephrologists. The therapy is based on symptomatic management involving the treatment of coexisting diseases (for instance, liver or kidney disorders) and acute conditions (such as hypoglycemia, seizures, metabolic acidosis) in hospital settings [14]. Patients experiencing acute metabolic decompensation should be admitted to the intensive care unit and initiated on intravenous glucose (dextrose) administration, treatment for metabolic acidosis (if present), and supportive care. Strict avoidance of fructose, sucrose, and sorbitol in the diet, along with supplementation of other carbohydrate sources (such as glucose, cornstarch), leads to rapid resolution of symptoms. The cornerstone of treatment is the implementation of appropriate dietary recommendations that involve restricting the intake of fructose, sucrose, sucralose, and sorbitol, thereby preventing the development of symptoms. To ensure long-term dietary compliance, repeated counseling sessions and clear instructions regarding dietary restrictions are essential [18].

Initial HFI symptoms overlap with other conditions, so recognizing specific signs is essential. A study by Úbeda et al. provides guidance on when to consider HFI. An algorithm assists in early HFI diagnosis. For patients with abdominal pain, vomiting, hypoglycemia after meals, aversion to sweets, and no cavities, food intake should be assessed. If fructose-containing foods were consumed, molecular diagnosis for HFI should be pursued. HFI is confirmed by detecting aldolase B gene mutations, particularly p.(Ala150Pro) and p.(Ala175Asp). If no mutations are found, a mixed food-tolerance test should be conducted to explore other pathologies. If fructose intake is unclear or minimal, and complications like growth retardation or liver issues are present, HFI suspicion increases, prompting molecular diagnosis. Otherwise, a mixed food-tolerance test should be conducted for further investigation [18].

## 4. Diet Therapy

The aim of the diet therapy is to eliminate fructose and disaccharides and polysaccharides from the diet, as fructose molecules (primarily sorbitol and sucrose) are released while digesting these. Currently, there are no conclusive data indicating a safe amount of fructose intake for the patient. This is partly due to the varying fructose tolerance depending on the residual activity of aldolase B enzyme. The problem lies in estimating the exact fructose content in foods and its actual consumption. Therefore, efforts should be made to maximize the reduction of that sugar in the diet, especially during the first two years of a child’s life [1].

In the diet of individuals with fructosemia, products containing fructose as well as sucrose and popular sweeteners such as sorbitol, inulin, xylitol, maltitol, or erythritol, are the most frequently excluded. This exclusion entails a significant reduction in the consumption of most fruits (except for avocado or small amounts of rhubarb), honey, sugar (sucrose), and products containing them. Another serious issue is the selection of medications in diseased states, as they may contain various sweetening agents (e.g., sorbitol) that constitute a source of fructose. Therefore, it is important to carefully review the composition of medications used. Among food products, particular attention should be paid to ready-made products, processed foods such as deli meats, sauces, spice blends, dietary supplements, but also toothpaste. The task of the physician overseeing the patient with fructosemia is to select a medication that will not be a source of fructose for the body [22].

Table 2 provides a comprehensive overview of banned and permissible products in the diet for patients with HFI.

Sugars such as glucose and lactose (milk sugar) as well as sweeteners like saccharin and aspartame (that have not been subjected to thermal processing) are safe for patients with hereditary fructose intolerance. White bread is preferred over wholegrain bread. This is due to the reduction in fructose content during the grain-refining process. Inadequate fiber content in the diet may cause constipation and dyspeptic symptoms; therefore, fiber supplementation should be considered [24].

Due to dietary restrictions, patients are also at risk of deficiencies in certain water-soluble vitamins, primarily vitamin C and folic acid. In the studies that have been conducted, statistically significant differences in serum vitamin C levels have been shown in healthy individuals and in those with fructosemia; therefore, daily supplementation is recommended. The same research results have not proven the need for folic acid supplementation. The authors, however, emphasize the need to conduct further research to establish optimal doses of vitamin C and the need for folic acid supplementation in patients with HFI (hereditary fructose intolerance) [25].

## 5. Residual Diagnostics

Periodic dietary restriction may manifest subtly as isolated hepatomegaly or sporadic elevation of aminotransferase activity [16]. Therefore, it is imperative to consider conditions associated with elevated liver enzyme activity, as outlined in Table 3, when evaluating potential hereditary fructose intolerance (HFI) in infants. A history of fructose consumption and the presence of hepatic steatosis are important clues suggesting hereditary fructose intolerance (HFI) in infants. Patients with HFI who are regularly fed a high-fructose diet may develop chronic liver disease in the form of hepatic steatosis, and even cirrhosis. Hence, during the differential diagnosis, consideration should be given to any conditions associated with elevated liver enzyme activity, hepatic steatosis, and cirrhosis.

**Figure 1:**
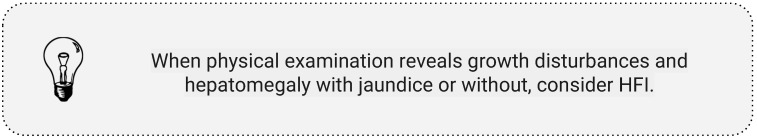
Consider HFI with growth disturbances and hepatomegaly.

In the physical examination, we may observe growth disturbances, which require diagnostic consideration in relation to celiac disease, anemia, nonspecific inflammatory bowel diseases, endocrinological causes (achondroplasia, acquired growth hormone deficiency, primary nutritional deficiency, intrauterine growth retardation, congenital hypothyroidism, congenital growth hormone deficiency), and genetic defects (e.g., Turner syndrome). Figure 1 emphasizes the importance of considering HFI when physical examination reveals growth disturbances and hepatomegaly, with or without jaundice.

Renal involvement typically manifests as proximal renal tubular acidosis and may lead to chronic kidney failure. During the differential diagnosis, one should consider inherited metabolic disorders (like cystinosis, Lowe syndrome, tyrosinemia, and Wilson disease) and systemic diseases (multiple myeloma, renal amyloidosis, and Sjögren’s syndrome) as most likely. Heavy metal poisoning (lead, cadmium, mercury), medications (acetazolamide, topiramate, tenofovir, lamivudine), conditions causing nephrocalcinosis (hyperparathyroidism, hypercalciuria), and sickle cell anemia should be taken into account, too [27].

Metabolic disturbances associated with HFI include hypoglycemia, lactic acidosis, hypophosphatemia, hyperuricemia, and hypermagnesemia [21]. Such symptoms may occur as a result of hormonal disorders such as congenital hyperinsulinism (channel disorders, metabolic disorders, transcription-factor disorders, syndromic disorders, congenital disorders of glycosylation), adrenal insufficiency, congenital pituitary insufficiency and growth hormone deficiency, congenital metabolic diseases (glycogen storage diseases, galactosemia, congenital disorders of gluconeogenesis, pyruvate carboxylase deficiency, phosphoenolpyruvate carboxykinase deficiency, glycerol kinase deficiency, congenital glycosylation disorders, disorders of fatty acid oxidation, ketone body metabolism disorders, oxidative phosphorylation disorders, and organic acidemias [1].

HFI is distinguished from other health conditions mainly by its manifestation of dominant gastrointestinal symptoms and a natural aversion to consuming sweets [1].

In contrast to the classical presentation of the aforementioned acute symptoms, some patients with residual enzymatic activity may remain asymptomatic or require higher fructose intake for symptoms to occur. HFI may also remain masked in the presence of concomitant diseases [28].

## 6. Discussion

Hereditary fructose intolerance (HFI) is a pathological condition resulting from a deficiency of the enzyme aldolase B. It is characterized by hypoglycemia, lactic acidosis, hypophosphatemia, hyperuricemia, hypermagnesemia, and hyperalaninemia caused by dysregulation of gluconeogenesis, glycogenolysis, and decreased levels of inorganic phosphates [4].

A significant challenge that occurs when diagnosing fructose intolerance is the nonspecific symptoms, which occur in many diseases of early childhood. So far, no genotype–phenotype correlation has been identified in patients with HFI. Symptoms only appear after exposure to fructose in the diet. This may occur either directly or indirectly through sucrose or sorbitol, substances which are commonly found in infant formulas. Therefore, unequivocal management cannot be easily determined. This is illustrated by the example of Li et al. [29]., who described four cases of acute liver failure in newborns and infants associated with multi-organ failure caused by common infant formula containing sucrose in patients with undiagnosed HFI. All patients were born at term after uncomplicated pregnancies and deliveries and were discharged from the hospital within the first week of life. There was no known consanguinity among them. One patient had an older brother in the family who died on the 28th day of life from a similar condition, although a specific diagnosis could not be established. Another patient had a maternal half-sister who required a liver transplant due to unspecified liver failure. Detailed dietary history was collected for all infants, although in two out of four cases, fructose exposure was unclear due to unreliable history or unclear labeling of ingredients, delaying the diagnosis. In all four cases, the newborn screening panel was normal. The diagnosis was confirmed by sequencing the ALDOB gene. All infants were homozygous for the common pathogenic variant c.448G > C (p.A150P) [29].

Strict adherence to a fructose-free diet is recommended during HFI treatment. However, it is debatable whether small amounts of fructose in the diet can be tolerated. Restricting fructose may lead to growth disturbances even in patients with HFI without clinical symptoms. There is insufficient information on the long-term effects of consuming minimal amounts of fructose. In a recent study conducted in Italy, a ten-year observation of patients with HFI was described. Hepatic steatosis (on ultrasound examination) persisted in 93.8% of patients despite them adhering to a fructose-restricted diet at a dose < 1.5 g/d [30]. The authors also found that a significant proportion of patients still had elevated transaminase activity (37.5%), even when adhering to the diet. There are two reasons for persistent liver dysfunction in patients with HFI. Firstly, fructose can be endogenously produced via the sorbitol–aldose reductase pathway, which may be activated after a meal enriched with glucose, administration of nephrotoxic drugs, or in stressful situations such as sepsis and major surgery. Secondly, permissible limits of fructose consumption may not be safe in asymptomatic patients with HFI.

This is confirmed by the determination of CDT (carbohydrate-deficient transferrin) by isoelectric focusing among patients with HFI on an FSS-free (fructose, sucrose, sorbitol) diet by Di Dato et al. [31]. A significant correlation was demonstrated between the amount of fructose consumed and the percentage of disialoTf and the ratio of tetrasialoTf/disialoTf. The authors suggested that the serum CDT profile could be considered a useful tool for monitoring FSS consumption. Additionally, CDT determination can be used to determine the maximum daily fructose tolerance in each patient with HFI. However, the main barrier to the use of this tool is the lack of widespread availability and high cost.

There are a lack of data in the literature on the long-term observation of patients with HFI. However, in a recent study of children with HFI, with a mean observation period of 10.3 ± 5.6 years, all children were asymptomatic, but most of them had hepatic steatosis and some had elevated transaminase activity [32]. Interestingly, fructose consumption in these children did not correlate with either outcome. Two case reports of HFI diagnosis in adulthood due to self-imposed fructose restriction since childhood may indicate that patients with HFI who strictly adhere to an FSS-free diet may have a good prognosis and a normal lifespan [33].

There is a need for research on the long-term outcomes of treating patients with HFI on an FSS-restricted diet to gain a fuller understanding of the consequences of NAFLD, cardiovascular diseases, and type 2 diabetes. However, the exact role of endogenous fructose production (via the polyol pathway) in intracellular F-1P accumulation remains to be determined in both animals and humans. HFI has diverse symptoms involving gastrointestinal, hepatic, and renal issues. It resembles many metabolic disorders that present similarly. Apart from genetic testing, there are no reliable laboratory markers that effectively diagnose this disease. A simple FSS-free diet usually leads to a good long-term prognosis. However, there is considerable controversy regarding the impact of dietary therapy on liver, biochemistry, and the coexistence of steatosis. Future research should be directed towards basic sciences and the long-term consequences of this disease.

Nucleic acid therapy offers some hope for treating this condition. This is known as gene therapy—a treatment that involves introducing foreign material (DNA or RNA) into cells. The nature or genetic information contained within the introduced DNA or RNA should exert a therapeutic effect. Mechanisms of action of introduced nucleic acids compel cells to produce protein encoded by the introduced gene. Thus, it is possible to produce proteins that are deficient or deficient in the body (e.g., in metabolic defects) [34]. However, the potential risk of complications and the high cost of therapy may discourage researchers from developing an effective preparation.

The emphasis placed on classical teaching regarding acute liver failure in children and biochemical disturbances, such as hypoglycemia and hypophosphatemia, following initial exposure to fructose may unintentionally increase the likelihood of overlooking cases of HFI characterized by other symptoms. Therefore, the index of suspicion must be high, and broad screening tests should be applied. HFI should be sought in every patient with unexplained causes of developmental delay. HFI is also often misdiagnosed in the case of other non-genetic and genetic disorders, including eating disorders, recurrent hepatitis, and glycogen storage disease. Moreover, fructose intolerance may not be pathognomonic for HFI alone, considering descriptions of rare patients with fruit and food protein-induced enterocolitis syndrome. Furthermore, the lack of a specific and practical biomarker for HFI means that neither newborn screening nor biochemical tests can be used to establish a diagnosis [30].

As of the publication of this article, no clinical studies on HFI are being conducted worldwide.

In the diet of individuals with hereditary fructose intolerance (HFI), vegetable juices (with certain exceptions) and nuts are permitted. The parents of the described child reported gastrointestinal discomfort and abdominal pain following the consumption of various vegetables. As plant-based products, vegetables contain varying amounts of fructose and sucrose, depending on the variety, cultivation methods, fertilizers used, and storage conditions. Patients with HFI may react differently to specific vegetables, necessitating caution in their consumption or, if possible, their elimination from the diet. Continuous dietary supervision by a nutritionist is essential to identify completely safe foods, those with questionable safety indices, and those that are entirely prohibited [1,34].

The takeaway lesson of this case is as follows: *Hereditary fructose intolerance (HFI) is a rare autosomal recessive disorder resulting from mutations in the aldolase B enzyme. Exposure to substances such as fructose, sucrose, and sorbitol leads to the accumulation of fructose-1-phosphate and its subsequent consequences. Common symptoms include gastrointestinal disturbances, aversion to sweets and fruits, and hypoglycemia. Hepatic manifestations include asymptomatic elevations in aminotransferase activity and hepatic steatosis. Genetic testing is preferred over measuring aldolase B activity in a liver biopsy sample to confirm the diagnosis. The cornerstone of managing HFI is the complete avoidance of foods containing fructose, sucrose, and sorbitol (FSS). Numerous challenges exist regarding tolerance levels, dietary restrictions, and the occurrence of fatty liver disease. Patients with HFI who strictly adhere to an FSS-free diet have an excellent prognosis and a normal life expectancy.*

## Figures and Tables

**Table 1:** Laboratory test results.

	Unit	25/02/23	28/02/23	01/03/23	03/03/23	06/03/23	13/03/23	04/04/23	29/05/23	29/11/23	12/12/23	23/02/24
AFP	ng/mL			7.95		8.36						
ALT	U/L	95	100	78	78		132	93	62	184	78	68
AST	U/L		99	72	75	84	100	72	53	136	58	55
GGTP	U/L		64	55	56	52	57	45	24	36		
ALP	U/L					212			174			
glucose	mg/dL	79		86		62		73	79			
CRP	mg/L	2.4		1.67					5.45	2.3		
direct bilirubin	mg/dL					0.21				0.13		
total bilirubin	mg/dL			0.5		0.62			0.2	0.34		
INR					1.04	0.96				1.05		
APTT	s				22.5							
total cholesterol	mg/dL					215		147	158			
chlorides	mmol/L								106			
inorganic phosphorus	mg/dL					6.01			5.05			
magnesium	mg/dL								2.21			
potassium	mmol/L			4.03					4.63			
sodium	mmol/L			139					139			
calcium	mmol/L			2.73		2.67			2.27			
creatinine	mg/dL			0.2					0.11			
TSH	μU/L					1.49			2.53			
fT4	ng/dL					1.14			1.07			
uric acid	mg/dL								4.7			
serum lactic acid	mmol/L								1.36			
IgA anti-tTG	U/mL								158.1			
anti-CMV IgG				negative								
anti-CMV IgM				negative								
HbS antigen					negative							
EBV VCA IgM				negative								
anti-HCV					negative							
stool parasites			negative						negative			
GSA-65 in stool									negative			

Abbreviations: AFP, Alpha-fetoprotein; ALT, Alanine aminotransferase; AST, Aspartate aminotransferase; GSA-65 Giardia Specific Antigen; CRP, C-reactive protein; anti-CMV IgG, anti-Cytomegalovirus IgG; anti-CMV IgM, anti-Cytomegalovirus IgM; APTT, Activated Partial Thromboplastin Time; EBV VCA IgM, Epstein-Barr Virus Viral Capsid Antigen IgM; ALP, Alkaline phosphatase; GGTP, Gamma-glutamyl transferase; TSH, Thyroid Stimulating Hormone; IgA anti-tTG, IgA anti-tissue transglutaminase; anti-HCV, Hepatitis C virus antibody; fT4, free thyroxine; INR, International Normalized Ratio.

**Table 2:** Banned and permissible products in the diet for the patients with fructosemia [1,23].

Banned	Permissible
Fruit, fruit juice, smoothie, cocktails, dried fruit	Vegetable juice, all vegetables except for sweet potatoes, green peas, zucchini
Sweetened dairy products	Dairy products with no added sugar (e.g., natural yogurt)
Processed meat products (e.g., frankfurters, ham)	Unprocessed meat, unprocessed fish
All and any sweets, pastry with sugar	Dietary sweets
Ketchup, jam, honey, sauces sweetened with sugar, sparkling drinks	Eggs, nuts

**Table 3:** Conditions associated with elevated liver enzyme activity [26].

Metabolic disorders and nutritional factors	- Overfeeding and obesity, metabolic syndrome, and NAFLD. - Starvation; protein malnutrition (kwashiorkor). - Diabetes. - Adrenal cortex hyperfunction. - Zinc deficiency. - Hyperlipidemia.
Digestive and absorptive disorders	- Pancreatic diseases (including cystic fibrosis). - Short bowel syndrome (after intestinal resections). - Celiac disease. - Nonspecific inflammatory bowel diseases (ulcerative colitis, Crohn’s disease).
Other inborn errors of metabolism	- Abetalipoproteinemia. - Storage diseases: sphingomyelin (Niemann–Pick disease), gangliosides (Tay–Sachs disease), glucocerebrosides (Gaucher disease), copper (Wilson’s disease), iron (hemochromatosis), glycogen (glycogen storage diseases), tyrosine, homocysteine, phytanic acid (Refsum syndrome), galactosemia, disorders of β-oxidation of fatty acids, α1-antitrypsin deficiency, protein glycosylation disorders; deficiency of acidic lysosomal lipase; inborn errors of urea cycle).
Infectious diseases	- Viral hepatitis (type C, type D, possibly type B in unvaccinated individuals). - Endotoxemia. - HIV infection.
Impact of hepatotoxic substances	- Drugs, especially glucocorticoids, antibiotics, cytostatics, vitamins (vitamin A in high doses) and others (salicylates, sodium valproate, warfarin). - Cocaine, ecstasy, and alcohol (in older children). - Toxins found in mushrooms (α-amanitin) [26].

## Data Availability

No new data were created or analyzed in this study. Data sharing is not applicable to this article.
